# AI-enhanced multi-timescale optimization strategy for virtual power plants: Advancing losad forecasting and dynamic demand response integration

**DOI:** 10.1371/journal.pone.0339606

**Published:** 2026-01-23

**Authors:** Guojun Xu, Guangjie Yang, Jie Bao, Huibo Feng, Feifei Zhang, Hua Zheng

**Affiliations:** 1 State Grid Handan Power Supply Company, Handan, China; 2 State Grid Hebei Electric Power Co., Ltd, Shijiazhuang, China; 3 the Department of Electrical and Electronic Engineering, North China Electric Power University, Beijing, China; King Fahd University of Petroleum & Minerals, PAKISTAN

## Abstract

The integration of renewable energy sources (RESs) introduces significant challenges related to uncertainty and intermittency in power grids. While Artificial Intelligence (AI) offers promising solutions for Virtual Power Plants (VPP) optimization, existing approaches often treat load forecasting, system dispatch, and demand response as loosely coupled components, limiting their ability to holistically manage these deep uncertainties. To address this, we propose a novel AI-enhanced multi-timescale optimization strategy that creates a synergistic, integrated framework. Methodologically, the approach begins with an attention-augmented Bidirectional Long Short-Term Memory (BiLSTM) model that generates high-fidelity spatiotemporal load forecasts, providing crucial spatial-aware inputs often overlooked by traditional models. These enhanced forecasts are then leveraged by a Model Predictive Control (MPC) strategy for more robust and proactive day-ahead and intraday dispatch. Crucially, the framework integrates a dynamic demand response (DDR) mechanism that is directly coupled with real-time MPC outputs, ensuring that load flexibility is mobilized based on immediate system needs rather than static signals alone. Simulations, driven by real-world operational data, confirm that this integrated strategy not only reduces operational costs and improves forecasting accuracy but also establishes a more resilient and adaptive VPP operational paradigm compared to prior AI-based methods.

## 1 Introduction

Energy security and the transition to a low-carbon future are fundamental to the evolution of modern power systems. As countries strive to achieve global carbon neutrality, the power sector is pivotal in supporting these ambitions. The large-scale integration of renewable energy sources (RESs), such as wind and photovoltaic (PV) systems, is essential to these objectives. However, the intermittent and variable nature of RESs has introduced significant uncertainties in the power supply-demand balance, complicating the stability and economic efficiency of power system operations [1,2].

To address these challenges, Virtual Power Plants (VPP)—which aggregate distributed energy resources (DERs), flexible loads, and energy storage systems—have emerged as a strategic solution. By optimizing the integration of RESs and managing system dispatch, VPP play a critical role in enhancing grid stability and operational efficiency, contributing to the realization of smarter, more resilient energy systems [3,4].

Artificial Intelligence (AI) techniques, including deep learning, reinforcement learning, and other advanced machine learning methods, offer promising solutions for managing the complexities posed by high RES penetration [5]. AI enables smarter operations in renewable energy-integrated power systems through improved real-time monitoring, load forecasting, and adaptive control. However, significant gaps remain in the use of AI for key tasks such as load forecasting, VPP scheduling, and dynamic demand response (DDR). However, despite these advancements, a critical gap persists in the literature: the majority of AI-based VPP studies apply sophisticated algorithms to individual sub-problems—such as forecasting, scheduling, or demand response—without creating a methodologically coherent, integrated framework. This siloed approach results in suboptimal performance, as forecasting errors are not effectively mitigated by scheduling, and demand response is not dynamically aligned with real-time operational constraints. The primary contribution of this work is to bridge this gap by introducing a holistic, AI-Enhanced optimization architecture.

Historically, load forecasting has evolved from statistical models like Autoregressive Integrated Moving Average (ARIMA) [6] to deep learning architectures. Early applications of deep learning saw studies proposing Long Short-Term Memory (LSTM) networks, which demonstrated exceptional performance in capturing long-term temporal dependencies in load data [7]. However, these models typically process aggregated load sequences and inherently overlook the critical spatial heterogeneity within VPP operational areas [8]. For instance, load responses to meteorological changes may differ significantly between urban and rural areas—a spatial correlation that purely temporal models fail to capture [9]. Recent advances have attempted to address this issue by incorporating spatial information. For example, some studies have used Graph Neural Networks (GNNs) to model physical grid topologies [10], while others have employed Convolutional Neural Networks (CNNs) to extract features from geographic data [11]. Although these approaches represent significant progress, they often treat spatial and temporal feature extraction as separate stages, potentially failing to capture their dynamic coupled interactions [12]. Additionally, methods focusing solely on uncertainty quantification, such as interval forecasting [13] and quantile regression [14], while improving robustness, do not address the fundamental issue of spatiotemporal feature fusion [15]. This leaves a critical gap for a model capable of comprehensively learning these deeply intertwined spatiotemporal dynamics, which serves as the primary motivation for our proposed attention-enhanced Bidirectional Long Short-Term Memory (BiLSTM) architecture.

In VPP dispatch, the paradigm has shifted from static day-ahead planning to dynamic multi-timescale optimization to better accommodate the volatility of RESs [16]. Model Predictive Control (MPC) has become a cornerstone of this approach, enabling rolling-horizon optimization that utilizes real-time data to correct day-ahead schedules [17]. For instance, proposed multi-timescale frameworks can effectively coordinate different temporal resolutions [18]. However, a key limitation of many such frameworks is their reliance on simple point forecasts or basic uncertainty models [19], which forces the MPC controller to be reactive—primarily correcting errors after they are observed—rather than proactively anticipating them. While some studies have integrated stochastic optimization or robust optimization to handle forecast errors [20], these methods can lead to overly conservative or computationally intensive solutions. The fundamental shortcoming lies in the inability to leverage the rich predictive information latent within high-dimensional spatiotemporal data [21]. Existing MPC applications for VPP [22] are not designed to ingest and act upon the nuanced, geographically-aware forecasts that modern AI can provide. Our work bridges this critical gap by designing an MPC framework explicitly tailored to the spatiotemporal predictions of the BiLSTM model, thereby enabling more proactive and cost-effective dispatch.

DDR is a crucial resource for VPP, but its practical effectiveness is often hindered by disconnection from system operations [23]. Advanced techniques such as Multi-Agent Reinforcement Learning (MARL) [24] and Evolutionary Game Theory [25] have produced sophisticated models of consumer behavior in response to price signals. However, a fundamental limitation of these approaches lies in the nature of the signals themselves. Typically, demand response (DR) programs are triggered by day-ahead price forecasts or predetermined peak periods [26], which are static and cannot adapt to sudden, intraday events such as unexpected drops in PV generation or transmission line constraints. For instance, proposed MARL frameworks optimize bidding strategies based on market prices but lack direct feedback loops from the physical state of VPP assets [27]. This temporal and information gap can lead to suboptimal or even counterproductive load shifts, as system operators are unable to precisely mobilize flexibility where and when it is most needed [28]. This highlights the urgent need for DR mechanisms that are not only price-sensitive but also integrated with dispatch. Our work addresses this issue by proposing a closed-loop DDR strategy, where DR signals are generated directly from the real-time outputs of the daily MPC scheduler, ensuring synergistic alignment between demand-side flexibility and the VPP’s immediate operational needs.

### 1.1 Contributions and innovations

To address these gaps, this paper proposes a novel multi-timescale VPP optimization strategy that integrates spatiotemporal feature-based load forecasting with dynamic DR. The key innovations of the proposed approach include:

1. The introduction of an attention-augmented BiLSTM model establishes a spatiotemporally-aware forecasting approach. Unlike conventional deep learning forecasters that only capture temporal dependencies, this model explicitly fuses spatial features. This provides the VPP scheduler with critical information on regional load heterogeneity, leading to more accurate and geographically nuanced dispatch plans.
2. Building upon the enhanced forecasts, a multi-timescale scheduling framework based on MPC is developed. This framework is specifically designed to leverage the rich spatiotemporal information from the forecasting model. This allows for proactive management of RES uncertainties, moving beyond the reactive adjustments common in existing MPC applications for VPP.
3. The core methodological advancement is a dispatch-integrated DDR mechanism that closes the loop between VPP dispatch and demand-side management. The proposed dynamic DR strategy is directly informed by the real-time outputs of the MPC scheduler. This overcomes the critical limitation of prior DR models, which often rely on static price signals and fail to align user responses with the immediate operational needs of the VPP, thereby achieving true supply-demand synergy.

The remainder of this paper is structured as follows: Section 2 describes the attention-augmented BiLSTM model for spatiotemporal load forecasting, detailing the network architecture and information flow. Section 3 presents the VPP system model, including mathematical formulations and constraints for DERs such as wind, PV systems, energy storage, and gas turbines. Section 4 introduces the multi-timescale optimization strategy, encompassing day-ahead economic dispatch, intraday rolling optimization, and dynamic DR. Section 5 validates the proposed strategies through simulation, analyzing forecasting accuracy, multi-energy flow balance, and intraday correction effectiveness. Finally, the conclusions and future research directions are presented.

## 2 Enhanced bidirectional LSTM load spatiotemporal prediction model integrated with attention mechanisms

The dynamic coupling of spatial and temporal features in power load data reveals that: within the same season, sensitivity to temperature variations differs significantly across regions; within the same region, dominant load types and their variation patterns shift seasonally. Accurate prediction requires models capable of simultaneously capturing and integrating such spatiotemporal interactions.

Therefore, this paper proposes an enhanced bidirectional LSTM load forecasting model integrated with attention mechanisms, designed to achieve precise predictions by comprehensively considering spatiotemporal characteristics of the data.

### 2.1 Bidirectional long short-term memory network

Conventional LSTM neural networks represent a specialized form of Recurrent Neural Network (RNN). By incorporating gating mechanisms to regulate information flow—comprising forget gate, input gate, and output gate—they effectively capture long-term dependencies [29].

The three gating structures modify the cell state by selectively removing or adding information, thereby mitigating gradient vanishing/explosion issues. The initial step operates as follows:


$$f_{t}=\sigma (R_{f}\cdot [h_{t-1},x_{t}]+o_{f})$$ (1)

where $R_{f}$ and $o_{f}$ denote the weight matrix and bias term of the forget gate, respectively.

After the forget gate operation, the information stored in LSTM’s internal memory state is jointly determined by the input gate and output gate. The process begins with the input gate defining which new information to update, expressed as follows:


$$i_{t}=\sigma (R_{i}\cdot [h_{t-1},x_{t}]+o_{i})$$ (2)

where $i_{t}$ represents a scaling factor between 0 and 1. When its value is 1, it signifies complete updating of current information; conversely, 0 indicates no updating. Subsequently, the input gate generates a candidate vector through the tanh activation function:


$${\tilde{C}}_{t}=\tanh(R_{i}\cdot [h_{t-1},x_{t}]+o_{c})$$ (3)

Based on the aforementioned processes, the cell state is updated using the obtained $f_{t}$, $i_{t}$, and ${\tilde{C}}_{t}$, transitioning from the previous state to the current state as follows:


$$C_{t}=f_{t}*C_{t-1}+i_{t}*{\tilde{C}}_{t}$$ (4)

The final step in LSTM computes the current output using the updated cell state and the output gate’s activation function, expressed as follows:


$$Y_{t}=\sigma (R_{o}\cdot [h_{t-1},x_{t}]+o_{0})$$ (5)

$$h_{t}=Y_{t}*\tanh(C_{t})$$ (6)

However, traditional unidirectional neural networks predict power load parameters by processing time-series data in a purely forward direction. This training approach makes inefficient use of the data and fails to fully capture its underlying characteristics. To address this, our paper proposes an attention-based BiLSTM model. Its bidirectional architecture allows information from both past and future hidden states to be recursively processed, enabling a more thorough discovery of the intrinsic relationships between the current load data and data from preceding and subsequent time steps. This ultimately enhances both the model’s prediction accuracy and its data utilization efficiency.

**Fig 1:**
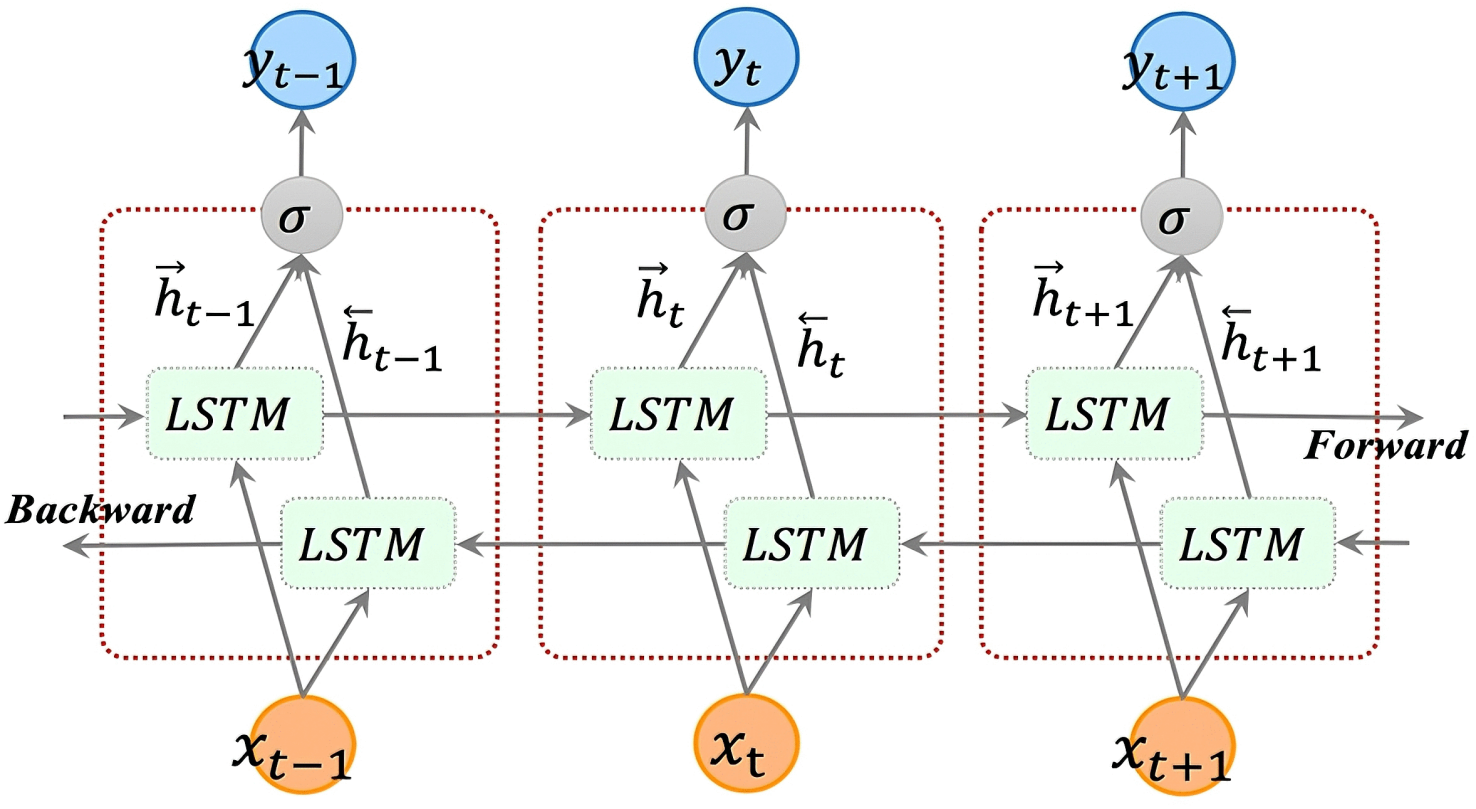
BiLSTM network architecture.

As illustrated in Fig 1, the BiLSTM network, in contrast to a standard LSTM, combines both a forward and a backward recurrent structure. While a conventional LSTM processes data unidirectionally from the past to the future, a BiLSTM introduces an additional, independent data flow from the future to the past. As a result, the BiLSTM is better able to capture the temporal dependencies within the data.

For the BiLSTM network proposed in this paper, the hidden state at each level is synthesized from three components: Forward-propagated hidden state from the previous timestep along the temporal axis $h_{t-1}$. Reverse-propagated hidden state from the subsequent timestep along the temporal axis $h_{i-1}$. Input vector at the current timestep $x_{t}$.

This combinatorial process of hierarchical hidden states is mathematically repre sented by Equation (7):


$$\{\begin{array}{c} {}*20lh_{t}=LSTM\left(x_{t},h_{t-1}\right)\\ h_{i}=LSTM\left(x_{t},h_{i-1}\right)\\ h_{t}={𝐚}_{t}h_{t}+{𝐛}_{t}h_{i}+{𝐜}_{t} \end{array}$$ (7)

where LSTM(·) represents computational procedure of standard LSTM. $h_{t}$ represents forward hidden state. $h_{i}$ represents backward hidden state. ${𝐚}_{t}$ and ${𝐛}_{t}$ represents Weight matrix for forward propagation and backward propagation. ${𝐜}_{t}$ is Bias term for current hidden layer.

### 2.2 Transformer attention mechanism

Based on the aforementioned BiLSTM architecture, this paper integrates a self-attention mechanism to capture dependencies among different timesteps in the sequence, comprising three core components: Query (Q), Key (K), and Value (V).


$$\{\begin{array}{c} {}*20cQ_{t}=W_{Q}\cdot h_{t}\\ K_{t}=W_{K}\cdot h_{t}\\ V_{t}=W_{V}\cdot h_{t} \end{array}$$ (8)

where $Q_{t}$, $K_{t}$ and $V_{t}$ denote the query vector, key vector, and value vector respectively; $W_{Q}$, $W_{K}$ and $W_{V}$ represent their corresponding weight matrices.

Building upon this foundation, further introduce an attention score computation mechanism. The calculation formula is defined as follows:


$$A=(\frac{q_{t}k_{t}^{T}}{\sqrt{d_{k}}})$$ (9)

where the normalization function softmax(·) computes attention weights. The calculation formula for attention weights can be determined by the following equation:


$$\alpha_{t}=\frac{\exp[A_{ij}]}{\sum {\mathrm{\backslash nolimits}}_{k=1}^{n}\exp[{A_{i}}_{k}]}$$ (10)

This weight primarily indicates the degree of influence from other inputs when computing outputs at different positions. Finally, perform weighted summation on the value vectors using attention weights:


$$z_{t}=\sum {\mathrm{\backslash nolimits}}_{i=1}^{n}\alpha_{ij}v_{j}$$ (11)

The result of the entire self-attention mechanism can be obtained, which can be described as follows:


$$Z=Attention(Q_{t},K_{t},V_{t})=soft\max(\frac{Q_{t}K_{t}^{T}}{\sqrt{d_{k}}})V_{t}$$ (12)

To enhance the expressive power of the model, this paper also employs a multi-head attention mechanism. By performing parallel computation of multiple attention heads, each head independently learns distinct representation spaces, thereby capturing different aspects of information within the sequence. Finally, the outputs of all heads are concatenated and linearly transformed to obtain the final attention result.

**Fig 2:**
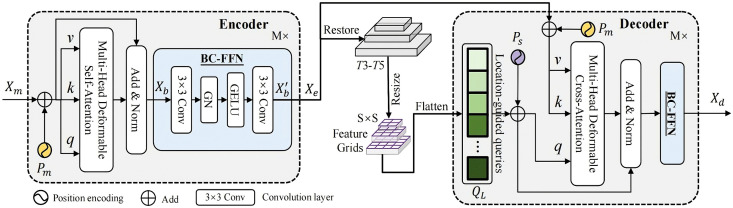
BiLSTM network architecture.

The overall structure of the Transformer attention mechanism designed in this paper can be shown in Fig 2 below, mainly consisting of multiple encoders and decoders stacked together. Each encoder and decoder layer contains multi-head attention mechanisms and corresponding feedforward neural networks.

Each encoder layer mainly consists of two sub-layers: a multi-head self-attention mechanism for capturing dependencies between different positions in the sequence, and a feedforward neural network for performing non-linear transformations on representations at each position. Each sub-layer is followed by residual connections and layer normalization, which can be described as:


Output=LayerNorm(X+Sublayer(X)) (13)

where Sublayer(X) denotes the multi-head attention mechanism or feedforward neural network.

The decoder layer resembles the encoder layer but incorporates a third sub-layer—a masked multi-head attention mechanism. This layer is crucial as it prevents the model from “looking ahead” at future information in the target sequence during the generation process, thereby ensuring prediction integrity. As with the encoder, each sub-layer is followed by a residual connection and layer normalization.

The feed-forward network (FFN) is a simple, two-layer fully-connected network. It typically employs the Rectified Linear Unit (ReLU) as its activation function, which is defined by the following expression:


$$FFN(x)=\max(0,xW_{1}+b_{1})W_{2}+b_{2}$$ (14)

where $W_{1}$ and $W_{2}$ denote weight terms, respectively.

The Transformer architecture, lacking recurrence or convolution, has no inherent understanding of sequence order. Therefore, positional encodings must be injected to provide the model with information about the relative or absolute position of each token. These encodings are generated using the following sine and cosine functions:


$$PE_{pos,2i}=\sin(\frac{pos}{10000^{\frac{2i}{\lambda_{model}}}})$$ (15)

$$PE_{pos,2i+1}=\cos(\frac{pos}{10000^{\frac{2i}{\lambda_{model}}}})$$ (16)

where pos denotes the token’s position in the sequence.

### 2.3 Fused attention mechanism BiLSTM load forecasting model

**Fig 3:**
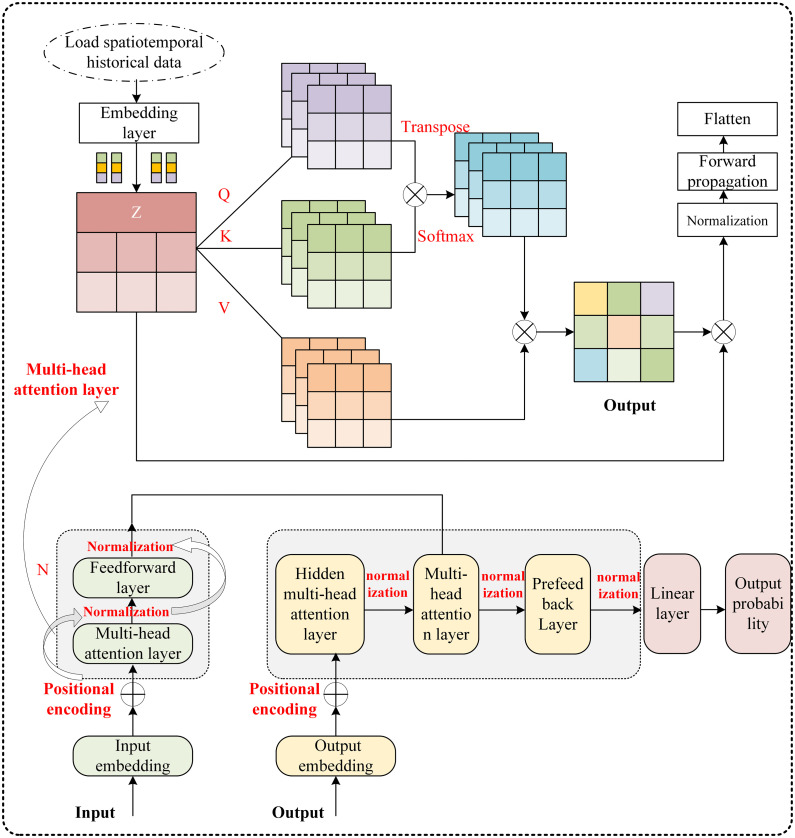
Fused attention mechanism BiLSTM load forecasting model.

This paper introduces an Attention-based BiLSTM model for load forecasting, with its overall architecture illustrated in Fig 3. The model is designed to capture the deep, coupled relationships between spatio-temporal features by integrating a BiLSTM network with an attention mechanism.

The model takes two primary inputs: the historical load sequence and a spatio-temporal feature matrix. This feature matrix is constructed to encompass both seasonal and regional dimensions. These inputs are concatenated to form an enhanced feature vector

$X=[X^{L};X^{ST}]\in {\mathbb{R}}^{T\times (1+d)}$, which is then fed into the BiLSTM layer for the extraction of high-level spatio-temporal features:


$$H=BiLSTM\mathrm{\backslash nolimits}(X),\;\;\;\;H\in {\mathbb{R}}^{T\times m}$$ (17)

The output of this layer simultaneously incorporates both the dynamic temporal characteristics of load and implicit spatial correlation information. In the spatiotemporal attention fusion layer, the weight distribution is generated through scaled dot-product attention:


$$A=softmax\mathrm{\backslash nolimits}\left(\frac{\left(HW^{\;\;Q}\right){\left(HW^{\;\;K}\right)}^{T}}{\sqrt{d_{k}}}\right)$$ (18)

then weighted fusion vector:


$$C=A\cdot \left(HW^{\;\;V}\right)$$ (19)

This process enables focusing on key time points along the temporal dimension, dynamically assigning weights across feature dimensions, and modeling spatiotemporal coupling. The final prediction output layer generates prediction results through a fully connected network:


$${\hat{Y}}_{t+1:t+\tau }=FC(C)$$ (20)

where τ is the prediction step size. Model training employs the mean squared error loss function:


$$ℒ=\frac{\text{1}}{N}\sum_{i=1}^{N}\sum_{j=1}^{\tau }{\left(y_{i,j}-{\hat{y}}_{i,j}\right)}^{2}$$ (21)

The model features a dynamic optimization mechanism, where attention weights adaptively adjust to seasonal patterns and differentially focus on unique regional characteristics. The model is trained end-to-end via backpropagation, with all parameters optimized jointly. To prevent overfitting and enhance generalization in complex spatio-temporal scenarios, an early stopping strategy is employed: training is halted if the validation loss does not improve for 10 consecutive epochs. The complete architecture, depicted in Fig 3, illustrates the entire information flow—from the input layer, through the BiLSTM for feature extraction and the attention mechanism for weighting, to the final prediction output.

## 3 Multi-energy complementary VPP model

**Fig 4:**
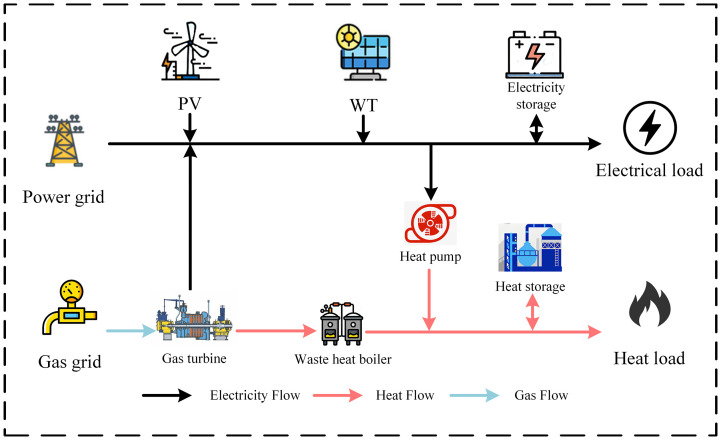
Multi-Energy complementary VPP model.

The multi-energy complementary VPP proposed in this study comprises a range of DERs, including wind turbines (WT), PV panels, a micro-gas turbine (GT), a Water Heater Boiler (WHB), a heat pump(HP), a heat storage tank, a battery energy storage system (BESS), and both thermal and electrical loads. The overall system architecture is depicted in Fig 4.

The indicator is the greenhouse gas emissions corresponding to producing one kilowatt-hour of electricity. For substations, carbon emissions originate from two phases: the construction phase and the operational phase. Carbon emissions per kilowatt-hour can be expressed as:


$$Q_{GT}=\frac{P_{GT}(t)\cdot \eta_{power}}{\eta_{hot}}$$ (22)

$$V_{GT}=\sum_{t=1}^{T}\left[\frac{P_{GT}(t)}{\eta_{power}L_{gas}}\cdot \Delta t\right]$$ (23)

$$k_{GT}=\frac{\eta_{hot}}{\eta_{power}}$$ (24)

where $P_{GT}(t)$ is the power generation output of the micro GT during time period *t* $Q_{GT}$ is the thermal power output of the GT during time period *t*. $\eta_{power}$ denotes the power generation efficiency; $\eta_{hot}$ is the thermal efficiency coefficient; $V_{GT}$ represents the volume of gas consumed by the micro GT for power generation; $L_{gas}$ is the lower heating value (LHV) of natural gas, typically fixed at 9.7 kWh/m^3^.

The GT operates as a CHP unit. The high-temperature exhaust gas it produces is captured by WHB to meet the thermal load. The mathematical model for this heat generation process is given by:


$$H_{GT\_t}=P_{GT\_t}/\delta_{GT}$$ (25)

$$H_{WHB\_t}=\lambda_{WHB}\cdot H_{GT\_t}$$ (26)

where $H_{GT\_t}$ is the thermal power output from the GT, $H_{WHB\_t}$ is the thermal power output from the WHB, $\delta_{GT}$ is the P2H ratio, and $\lambda_{WHB}$ is the heat recovery efficiency.

The HP functions as a P2H device, converting electricity into thermal energy to satisfy the microgrid’s heat demand. Its governing mathematical model and constraints are as follows:


$$\left\{ \begin{array}{c} Q_{HP}=\eta_{HP}\cdot P_{HP}\\ Q_{HP}\leq Q_{HP\_\max} \end{array}\right.$$ (27)

where $P_{HP}$ denotes the input electrical power of the HP, $\eta_{HP}$ is its coefficient of performance (COP), $Q_{HP}$ is its thermal power output, and $Q_{HP\_\max}$ is its maximum thermal power output.

This study employs a BESS. The dynamics of the battery are described by the following mathematical model, which tracks its State of Charge (SOC) over time [30]:


$$S_{Bat}(t)=\left\{ \begin{array}{c} S_{Bat}(t-1)\cdot (1-\sigma_{Bat})-\frac{P_{Bat}(t)\Delta t}{\eta_{dis}}\\ S_{Bat}(t-1)\cdot (1-\sigma_{Bat})-P_{Bat}(t)\Delta t\cdot \eta_{cha} \end{array}\right.$$ (28)

where $S_{Bat}(t)$ is the SOC of the battery at the end of time step *t* (kWh); $S_{Bat}(t-1)$ is the SOC at the end of the previous time step *t-1* (kWh); $P_{Bat}(t)$ is the power of the battery during time step *t* (kW), with a positive value indicating discharging and a negative value indicating charging; $\eta_{dis}$ and $\eta_{cha}$ are the charging and discharging efficiencies, respectively; $\sigma_{Bat}$ is the self-discharge rate; Δt is the duration of the time step.

The operational principle of the thermal storage tank is analogous to that of the BESS. It stores excess thermal energy (charges) when available and releases it (discharges) when direct heat generation is insufficient to meet the thermal load [30]. The operation is subject to the following capacity and power constraints:


$$\{\begin{array}{c} {}*20cW_{HS\_t}=W_{HS\_t-1}\left(1-\gamma_{h}\right)+\left(\eta_{HS\_chr}H_{HS\_chr\_t}-H_{HS\_dis\_t}/\eta_{HS\_dis}\right)\\ W_{HS\_\min}\leq W_{HS\_t}\leq W_{HS\_\max} \end{array}$$ (29)

$$\{\begin{array}{c} {}*20lH_{HS\_chr\_\min}\leq H_{HS\_chr\_t}\leq H_{HS\_chr\_\max}\\ H_{HS\_dis\_\min}\leq H_{HS\_dis\_t}\leq H_{HS\_dis\_\max} \end{array}$$ (30)

where $W_{HS\_t}$ is the amount of thermal energy stored in the HS at time *t*; $H_{HS\_chr\_t}$ and $H_{HS\_dis\_t}$ are the thermal charging (heat absorption) and discharging (heat release) powers at time *t*, respectively; $\eta_{HS\_chr}$ and $\eta_{HS\_dis}$ are the thermal charging and discharging efficiencies, respectively; $W_{HS\_\min}$ and $W_{HS\_\max}$ are the minimum and maximum storage capacity of the HS, respectively; $H_{HS\_dis\_\min}$ and $H_{HS\_dis\_\max}$ are the minimum and maximum thermal discharging power, respectively; $H_{HS\_chr\_\min}$ and $H_{HS\_chr\_\max}$ are the minimum and maximum thermal charging power, respectively.

## 4 Multi-time-scale optimal dispatch model for a VPP with DDR

**Fig 5:**
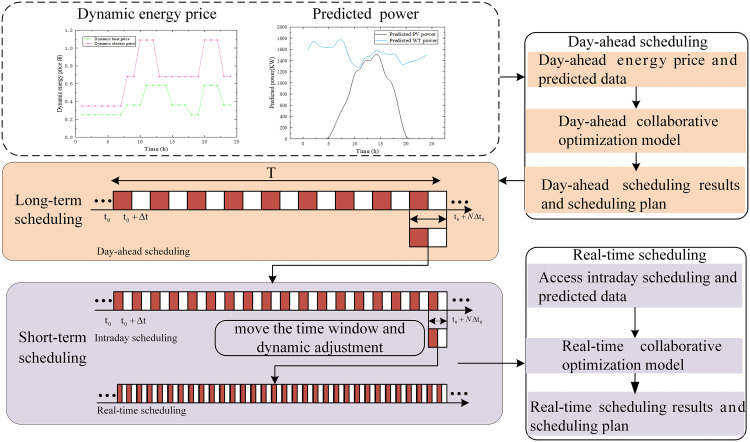
Multi-time-scale optimal dispatch model.

This study proposes a two-stage, multi-time-scale optimal dispatch framework based on MPC, which integrates day-ahead scheduling and intra-day rolling optimization. The MPC methodology operates by predicting the system’s future behavior at each control interval, solving a finite-horizon optimization problem over a prediction horizon p, and then implementing only the first element of the resulting control sequence. The architecture of this rolling optimization approach is illustrated in the Fig 5 below.

The day-ahead stage establishes an initial 24-hour dispatch plan with a 1-hour time resolution, based on day-ahead forecasts. Recognizing that the forecast accuracy for renewable generation (wind and solar) and load demand increases with finer time resolutions, a multi-time-scale approach is employed to refine this initial plan. The intra-day stage performs rolling optimization with a shorter 4-hour dispatch horizon and a 15-minute time resolution, continuously correcting the day-ahead schedule to adapt to real-time conditions.

### 4.1 Day-ahead dispatch stage

The objective function of the day-ahead optimal dispatch model is to minimize the total daily operating cost of the VPP, formulated as:


$$C_{Total}=\min\left(C_{OP}+C_{EN}+C_{Gird}+C_{DR}\right)$$ (31)

The total cost $C_{Total}$ comprises four components: the VPP’s operating $C_{OP}$ and the environmental cost $C_{EN}$, the cost of power exchange with the main grid $C_{Gird}$, and the DR compensation cost $C_{DR}$.

(1) Operating and Degradation Cost

This term includes the operational costs of the GT—encompassing fuel, start-up, and shutdown costs—and the degradation cost of the BESS due to cycling. It is calculated as:


$$C_{OP}=\sum_{t=1}^{T}\left|P_{Bat}(t)\right|K_{Bat}\Delta t+\sum_{t=1}^{T}\left(\gamma_{GT}\Delta t+C_{GT}^{on}S_{GT}^{on}(t)+C_{GT}^{off}S_{GT}^{off}(t)\right)$$ (32)

where $P_{Bat}(t)$ is the magnitude of the battery’s power flow (charging or discharging) at time *t*; $K_{Bat}$ is the battery degrada*t*ion cost coefficient; $\gamma_{GT}$ is the MT’s fuel cost function; $C_{GT}^{on}$ and $C_{GT}^{off}$ are the start-up and shutdown costs; $S_{GT}^{on}(t)$ and $S_{GT}^{off}(t)$ are binary variables representing the MT’s start-up and shutdown status at time *t*.

The MT’s fuel cost is typically a quadratic function of its power output:


$$\gamma_{GT}=aP_{GT}^{3}+bP_{GT}^{2}+cP_{GT}+d$$ (33)

where a, b, c and d are the fuel cost coefficients.

(2) Environmental Cost

This cost quantifies the environmental impact of emissions from the MT’s operation:


$$C_{EN}=\sum_{t=1}^{T}\left(P_{GT}(t)\cdot \Delta t\cdot K_{GT}^{EN}\right)$$ (34)

where $P_{GT}(t)$ is the MT’s power output, and $K_{GT}^{EN}$ is the unit emission cost coefficient.

(3) Grid Interaction Cost

This represents the cost of electricity transactions with the main utility grid:


$$C_{Gird}=\sum_{t=1}^{T}\left(P_{Grid}(t)\cdot \Delta t\cdot \sigma_{Grid}\right)$$ (35)

where $P_{Grid}(t)$ is the absolute power exchanged with the main grid, and $\sigma_{Grid}$ is the unit cost of grid power.

(4) DR Compensation Cost

This is the cost of compensating consumers for participating in the DR program by curtailing their loads:


$$C_{DR}=\lambda_{e}\sum_{t=1}^{T}\Delta L_{e,cut\_t}+\lambda_{h}\sum_{t=1}^{T}\Delta L_{h,cut\_t}$$ (36)

where $\lambda_{e}$ and $\lambda_{h}$ are the amounts of curtailed electrical and thermal load, respectively, and $\Delta L_{e,cut\_t}$ and $\Delta L_{h,cut\_t}$ are their corresponding unit compensation prices.

The day-ahead optimal dispatch is subject to the following operational constraints:

(1) Power Balance Constraints

At each time interval *t*, the VPP must maintain a balance between power generation and consumption for both electricity and heat.


$$P_{Load}(t)+P_{Bat\_Dis}(t)+P_{Buy}(t)=P_{Wt}(t)+P_{Pv}(t)+P_{Bat\_Cha}(t)+P_{GT}(t)+P_{Sell}(t)$$ (37)

$$H_{WHB\_t}+Q_{Hp\_t}+H_{HS\_dis\_t}=L_{h\_t}+H_{HS\_chr\_t}$$ (38)

where $P_{Load}(t)$ is the electrical loads. $P_{Wt}(t)$, $P_{Pv}(t)$ and $P_{GT}(t)$ are the power outputs from wind, PV, and the GT. $P_{Sell}(t)$ represent power purchased from and sold to the main grid. $P_{Bat\_Dis}(t)$ is the battery’s discharging powers. $L_{h\_t}$ is the thermal loads.

(2) Tie-Line Power Exchange Limits

The power exchanged with the main grid via the tie-line is constrained by its physical capacity:


$$P_{Gird}^{\min}\leq P_{Grid,t}\leq P_{Gird}^{\max}$$ (39)

where $P_{Gird}^{\min}$ and $P_{Gird}^{\max}$ define the allowable range for power import/export.

(3) BESS Constraints

Energy storage system operation is governed by power and energy SOC limits:


$$\{\begin{array}{c} {}*20l0\leq P_{Bat\_Cha}(t)\leq P_{Bat\_Cha,\max}\\ 0\leq P_{Bat\_Dis}(t)\leq P_{Bat\_Dis,\max}\\ P_{Bat\_Dis,\max}=\min\left\{\left(SOC^{\max}-SOC_{t-1}\right)\times C_{Bat}\times \eta_{dis},P_{Bat\_E}\right\}\\ P_{Bat\_Cha,\max}=\min\left\{\left(SOC^{\max}-SOC_{t-1}\right)\times C_{Bat}\times \frac{\text{1}}{\eta_{cha}},P_{Bat\_E}\right\} \end{array}$$ (40)

where $P_{Bat\_Cha}(t)$ and $P_{Bat\_Dis}(t)$ are the maximum charging/discharging power rat ings. $P_{Bat\_Cha,\max}$ and $P_{Bat\_Dis,\max}$ are binary variables to prevent simultaneous charging and discharging.


$$\{\begin{array}{c} {}*20lSOC_{\min}\leq SOC_{t}\leq SOC_{\max}\\ SOC_{star}=SOC_{end} \end{array}$$ (41)

(4) Renewable Energy Output Constraints

The power output from wind and solar resources is constrained by the forecasted availability.


$$0\leq P_{PV}(t)\leq N_{PV}P_{PV0}(t)$$ (42)

$$0\leq P_{WT}(t)\leq N_{WT}P_{WT0}(t)$$ (43)

where $P_{PV}(t)$ and $P_{WT}(t)$ represent the maximum available power from the wind farm and PV array at time *t*, respectively. $N_{PV}$ and $N_{WT}$ are the number of installed PV panels and WT. The terms $P_{Pv0}(t)$ and $P_{Wt0}(t)$ denote the forecasted power generation from a single unit of each technology at time *t*.

### 4.2 Intra-day dispatch stage

To address the deviations between the day-ahead dispatch schedule and actual real-time operation—which stem from forecast inaccuracies and unpredictable weather changes—this paper employs an intra-day rolling dispatch strategy. This strategy periodically adjusts the schedule using updated short-term forecasts for load, wind, and solar power.

To maintain the integrity of the day-ahead plan and prevent excessive adjustments, the objective of the intra-day optimization is to minimize the deviations from the pre-established day-ahead schedule. This is achieved by incorporating a penalty term for these adjustments into the objective function. Therefore, the intra-day objective is to minimize the sum of these penalty costs over the rolling dispatch horizon, as formulated in Equation (44):


$$C_{Total\_0}=\min\left(C_{punish\_g}+C_{punish\_h}\right)$$ (44)

where $C_{punish\_g}$ and $C_{punish\_h}$ are the penalty costs associated with the adjustments made to the power outputs of the electrical and thermal units, respectively.


$$C_{punish\_g}=\min\left[\Delta P_{Bat}\lambda_{bat}+\Delta P_{GT}\lambda_{gt}+\Delta P_{Gird}(t)\lambda_{gird}\right]$$ (45)

and


$$\left\{ \begin{array}{c} \Delta P_{Bat}=\sum_{t=1}^{T}\left|\left|P_{Bat}(t)\right|-\left|P_{Bat\_0}(t)\right|\right|\\ \Delta P_{GT}=\sum_{t=1}^{T}\left|P_{GT}(t)-P_{GT\_0}(t)\right|\\ \Delta P_{Gird}=\sum_{t=1}^{T}\left|P_{Gird}(t)-P_{Gird\_0}(t)\right| \end{array}\right.$$ (46)

In this equation, $\Delta P_{Bat}$, $\Delta P_{GT}$ and $\Delta P_{Gird}$ represent the total adjustments made to the power schedules of the battery, microturbine, and grid exchange during the intra-day stage, compared to the original day-ahead plan. These adjustments are weighted by their respective penalty coefficients, $\lambda_{bat}$, $\lambda_{gt}$, and $\lambda_{gird}$. Furthermore, $P_{Bat\_0}(t)$, $P_{GT\_0}(t)$, and $P_{Gird\_0}(t)$ denote the actual intra-day power outputs for the battery, microturbine, and grid interaction at time *t.*


$$C_{punish\_h}=\min\left[\Delta P_{HS}\lambda_{hs}+\Delta P_{WHB}\lambda_{whb}+\Delta P_{HP}(t)\lambda_{hp}\right]$$ (47)

$$\left\{ \begin{array}{c} \Delta P_{HS}=\sum_{t=1}^{T}\left|\left|P_{HS}(t)\right|-\left|P_{HS\_0}(t)\right|\right|\\ \Delta P_{WHB}=\sum_{t=1}^{T}\left|P_{WHB}(t)-P_{WHB\_0}(t)\right|\\ \Delta P_{HP}=\sum_{t=1}^{T}\left|P_{HP}(t)-P_{HP\_0}(t)\right| \end{array}\right.$$ (48)

where, $\Delta P_{HS}$, $\Delta P_{WHB}$, and $\Delta P_{HP}$ represent the total adjustments to the scheduled thermal output of the heat storage tank, WHB, and HP, comparing the intra-day plan to the day-ahead schedule. These adjustments are penalized using their respective coefficients, $\lambda_{hs}$, $\lambda_{whb}$, and $\lambda_{hp}$. Furthermore, $P_{HS\_0}(t)$, $P_{WHB\_0}(t)$ and $P_{HP\_0}(t)$ denote the intra-day dispatched thermal power for these units at time *t*.

The constraints for the WT, PV units, battery storage, and grid interaction are the same as in the day-ahead model and are thus omitted. For the shorter intra-day scheduling horizon, however, the microturbine’s ramping constraints are introduced as follows:


$$-R_{gt\_d}\leq P_{GT\_0}(t)-P_{GT\_0}(t-1)\leq R_{gt\_u}$$ (49)

where $R_{gt\_d}$ and $R_{gt\_u}$ represent the upper and lower limits of the microturbine’s ramping rate.

### 4.3 Dynamic demand response model

DDR refers to the modification of electricity consumption patterns by end-users in response to signals from the supply side, which are typically issued when electricity market prices are high or system reliability is compromised. Upon receiving incentive-based signals—such as notifications of price increases or offers of direct compensation for load reduction—users alter their conventional electricity usage strategies. This leads to a reduction or shift in their electricity load during specific periods. In essence, DR constitutes a beneficial interaction between consumers and the grid.

Different loads respond differently to the same price signals. Price-based DR loads are generally categorized into curtailable loads (CL) and shiftable loads (SL). A curtailable load determines whether to interrupt its consumption by evaluating the change in electricity price before and after the DR event.

The characteristics of DR are commonly described using a price elasticity matrix. The element $e_{m,n}$ in the *m*-th row and *n*-th column of the elasticity matrix E(m,n) represents the price elasticity coefficient of the load at time m with respect to the electricity price at time n. The mathematical model is as follows:


$$e_{m,n}=\frac{\Delta P_{L,m\_e}/P_{L,m\_e0}}{\Delta \rho_{n}/\rho_{n\_0}}$$ (50)

In the equation, $\Delta P_{L,m\_e}$ represents the change in load at time *m*, $P_{L,m\_e0}$ is the original load at time m, $\Delta \rho_{n}$ denotes the change in electricity price at time *n*, and $\rho_{n\_0}$ is the original price at time *n*.

Consequently, the change in the curtailable load after DR, $\Delta P_{CL,m\_e}$, is calculated as follows:


$$\Delta P_{CL,m\_e}=P_{CL,m\_e0}\left[\sum_{n=1}^{24}E_{CL}(m,n)\frac{\rho_{n}-\rho_{n\_0}}{\rho_{n\_0}}\right]$$ (51)

where $P_{CL,m\_e0}$ is the original amount of curtailable load at time *m*. $E_{CL}\left(m,n\right)$ is the price elasticity matrix for the curtailable load. $\rho_{n}$ is the electricity price at time *n*.

The principle of shiftable load is that users flexibly adjust their energy consumption timing based on their own needs and real-time electricity/heat price information. This allows loads from high-price periods to be shifted to low- or flat-price periods. The mathematical model for the change in shiftable load after DR is given by:


$$\Delta P_{SL,m\_e}=P_{SL,m\_e0}\left[\sum_{n=1}^{24}E_{SL}(m,n)\frac{\rho_{n}-\rho_{n\_0}}{\rho_{n\_0}}\right]$$ (52)

where $\Delta P_{SL,m\_e}$ is the original amount of shiftable load at time m. $E_{SL}\left(m,n\right)$ is the price elasticity matrix for the shiftable load.

## 5 Simulation results and analysis

To validate the effectiveness of the proposed multi-timescale optimization strategy for VPP, which integrates spatiotemporal feature-based load forecasting and DDR. Simulations of the constructed model were conducted using MATLAB R2021a with the CPLEX solver.

### 5.1 Objects case study data

**Fig 6:**
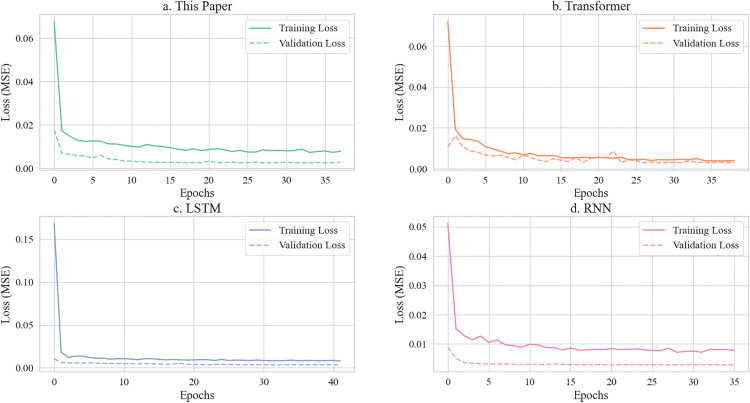
Model training and validation loss.

**Fig 7:**
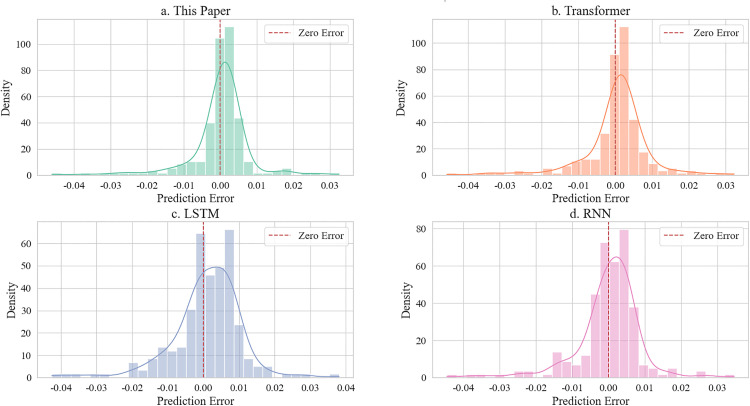
Error distribution analysis.

**Fig 8:**
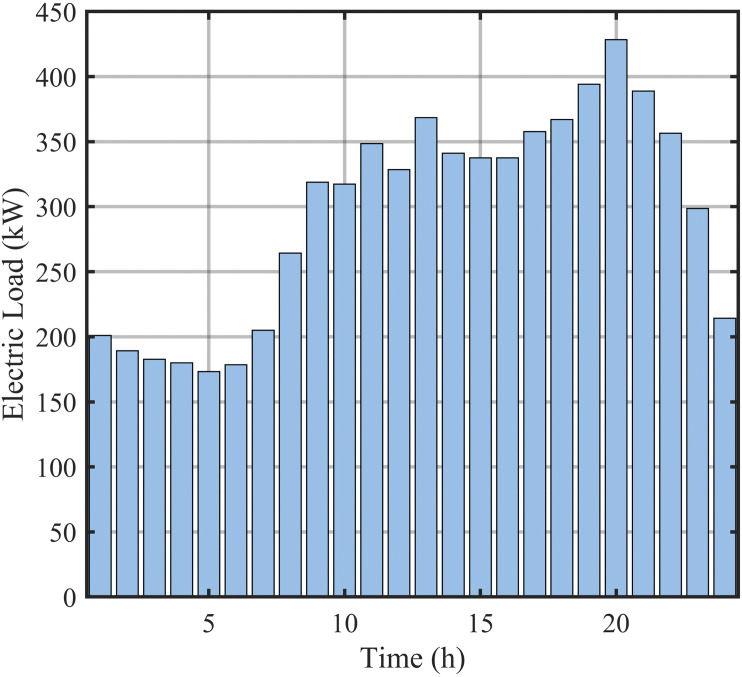
Electrical load forecasting data.

**Fig 9:**
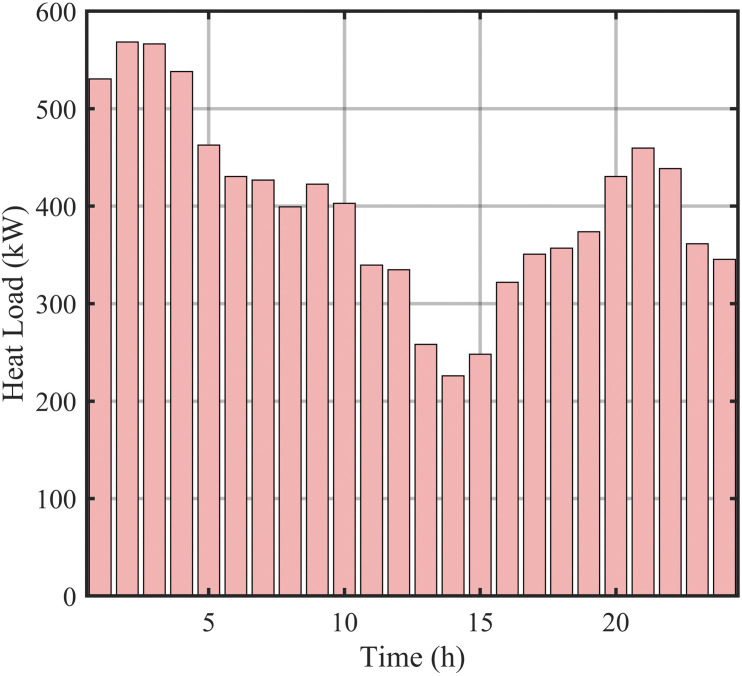
Heat load forecasting data.

To validate the effectiveness and practical applicability of the proposed multi-timescale optimization strategy, this study utilizes a comprehensive dataset of actual operational data from a regional multi-energy VPP. This dataset provides a realistic foundation for our simulation-based validation, ensuring that the model is tested against real-world dynamics and uncertainties. The data, spanning a three-year period with a 15-minute temporal resolution, includes historical time-series for electrical and thermal loads, wind and solar power generation, and market electricity prices. While the raw operational data is subject to a confidentiality agreement and cannot be publicly disclosed, the key technical parameters of the VPP components, derived from the real system’s specifications, are detailed in Table 1. The system is configured with a 520 kW wind turbine, a 300 kW photovoltaic array, a 400 kWh energy storage system with a 60 kW charge/discharge rating, a 300 kW micro GT, a 400 kW HP, and a 2000 kWh thermal storage tank. The peak electrical and thermal loads observed in the dataset are 440 kW and 570 kW, respectively. The time-of-use electricity/heat prices and grid tariffs used in the simulation, shown in Tables 2 and 3, are also based on the actual pricing schemes from the corresponding period. The day-ahead and intra-day forecast curves for renewable generation and loads, presented in Figs 6–9, are generated by our proposed model using this historical dataset.

**Table 1:** VPP system parameters.

Parameter Type	Value	Parameter Type	Value
Load shedding compensation coefficient	0.98	Battery charging efficiency	0.95
Tie-line power limit	200	Battery discharging efficiency	0.95
Heat pump heating efficiency	3	Initial SOC	0.5
Natural gas price	2.55	SOC upper limit	0.85
GT fuel consumption coefficient a	2.67	SOC lower limit	0.2
GT fuel consumption coefficient b	66.2	Heat storage efficiency	0.95
GT fuel consumption coefficient c	100	Heat release efficiency	0.90
GT electric-to-heat ratio	0.35	Initial Heat storage level	0.5
GT power upper limit	300	Maximum charge level	0.98
WHB heat recovery efficiency	0.85	Maximum discharge level	0.05

**Table 2:** Time-of-use electricity and heat prices.

Time	Electricity Price (¥/kWh)	Heating Price (¥/kWh)	Time	Electricity Price (¥/kWh)	Heating Price (¥/kWh)
1	0.35	0.25	13	0.68	0.58
2	0.35	0.25	14	0.68	0.58
3	0.35	0.25	15	0.68	0.36
4	0.35	0.25	16	0.68	0.36
5	0.35	0.25	17	0.68	0.36
6	0.35	0.25	18	1.09	0.25
7	0.35	0.25	19	1.09	0.25
8	0.68	0.36	20	1.09	0.58
9	0.68	0.36	21	1.09	0.58
10	1.09	0.36	22	0.68	0.58
11	1.09	0.58	23	0.35	0.36
12	1.09	0.58	24	0.35	0.36

**Table 3:** Grid electricity purchase/sale prices.

Time	Electricity Purchase Price (¥/kWh)	Electricity Sale Price (¥/kWh)
0:00 ~ 7:00,22:00 ~ 24:00	0.35	0.22
7:00 ~ 9:00,12:00 ~ 17:00,21:00 ~ 22:00	0.68	0.42
9:00 ~ 12:00,17:00 ~ 21:00	1.09	0.65

To ensure the reproducibility of our forecasting results, this section provides detailed information regarding the experimental setup for the proposed model. The historical operational data, including load and renewable energy generation profiles, was obtained from a regional VPP operator under a confidentiality agreement, as mentioned in the Data Availability Statement. The dataset spans a three-year period from January 1, 2021, to December 31, 2023, with a 15-minute temporal resolution.

The dataset was chronologically partitioned to prevent data leakage and ensure a robust evaluation of the model’s predictive capabilities. Data from January 1, 2021, to December 31, 2022 (approximately 70% of the total data) was allocated for the training set. Data from January 1, 2023, to June 30, 2023 (15%) served as the validation set, used for hyperparameter tuning and triggering the early stopping mechanism. The remaining data, from July 1, 2023, to December 31, 2023 (15%), was reserved as the final, unseen test set to evaluate the model’s generalization performance. Prior to model training, all input features were normalized to a range of [0, 1] using min-max scaling to improve convergence speed and stability. The key hyperparameters, selected through a combination of grid search and empirical validation on the validation set, are detailed in Table 4.

**Table 4:** Hyperparameters for the proposed forecasting model.

Hyperparameter	Value
Optimizer	Adam
Learning Rate	0.001
Batch Size	64
Max Epochs	1000
Early Stopping Patience	200 epochs
BiLSTM Layers	2
Hidden Units (per layer)	128
Multi-Head Attention Heads	8
Dropout Rate	0.2
Input Sequence Length	96 steps (24 hours)
Output Sequence Length	16 steps (4 hours)

Fig 6 compares the training convergence of four models: (a) the proposed attention-based BiLSTM, (b) Transformer, (c) LSTM, and (d) RNN. The proposed model (a) rapidly stabilizes within 20 epochs (MSE ≈ 0.015), with validation loss significantly lower than others. Although Transformer (b) converges quickly, its final validation loss (MSE ≈ 0.025) exceeds the proposed method. Traditional LSTM (c) and RNN (d) exhibit pronounced overfitting and higher stabilized loss (MSE > 0.03), demonstrating that integrating attention mechanisms with bidirectional structure enhances learning efficiency and generalization.

Fig 7 analyzes prediction error distributions. The proposed model (a) exhibits the most compact error concentration near zero (±0.01 range, peak density 0.04), indicating high accuracy and stability. Transformer (b) shows a sharp primary peak but a secondary peak at ±0.02, revealing sensitivity to outliers. LSTM (c) and RNN (d) display broad error spreads (±0.03) with lower density peaks (≤0.03), confirming that spatiotemporal feature fusion effectively controls prediction deviations.

Fig 8 displays electrical load forecasting curves, capturing spatiotemporal characteristics across seasons and regions. Fig 9 presents thermal load forecasting curves, reflecting periodic fluctuations in heating systems. Together, they provide the data foundation for multi-timescale scheduling.

### 5.2 Day-ahead optimal dispatch results analysis

Building upon the high-accuracy load forecasting data and incorporating the DDR model, this section presents an analysis of the day-ahead optimal dispatch results for the VPP.

1 Operational strategy analysis of energy storage systems

**Fig 10:**
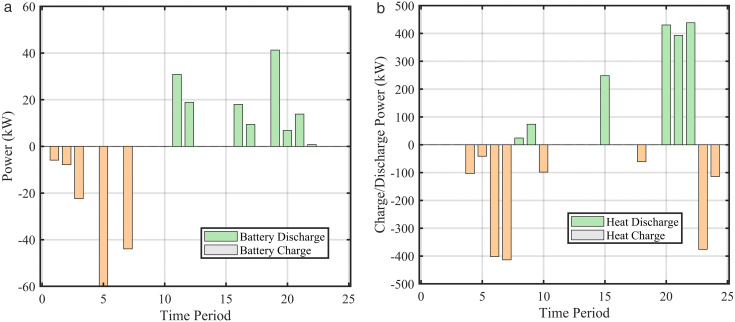
Energy storage system operation (a) Battery charge/discharge, (b) Heat storage charge/discharge.

**Fig 11:**
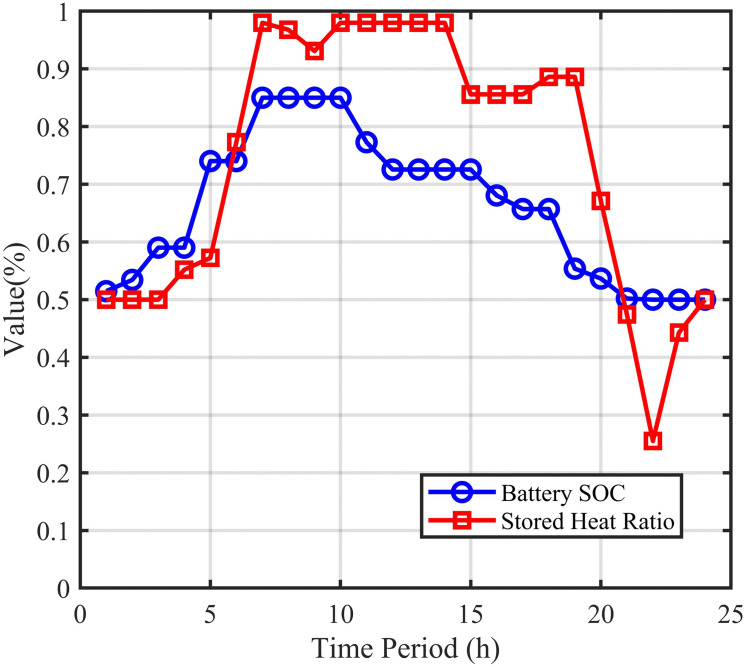
Average wind power output forecast.

To further explore the role of energy storage systems in optimal scheduling, Figs 10, and 11 are jointly presented to illustrate the dynamic charging/discharging power and SOC variations of electrical and heat storage systems.

**Fig 12:**
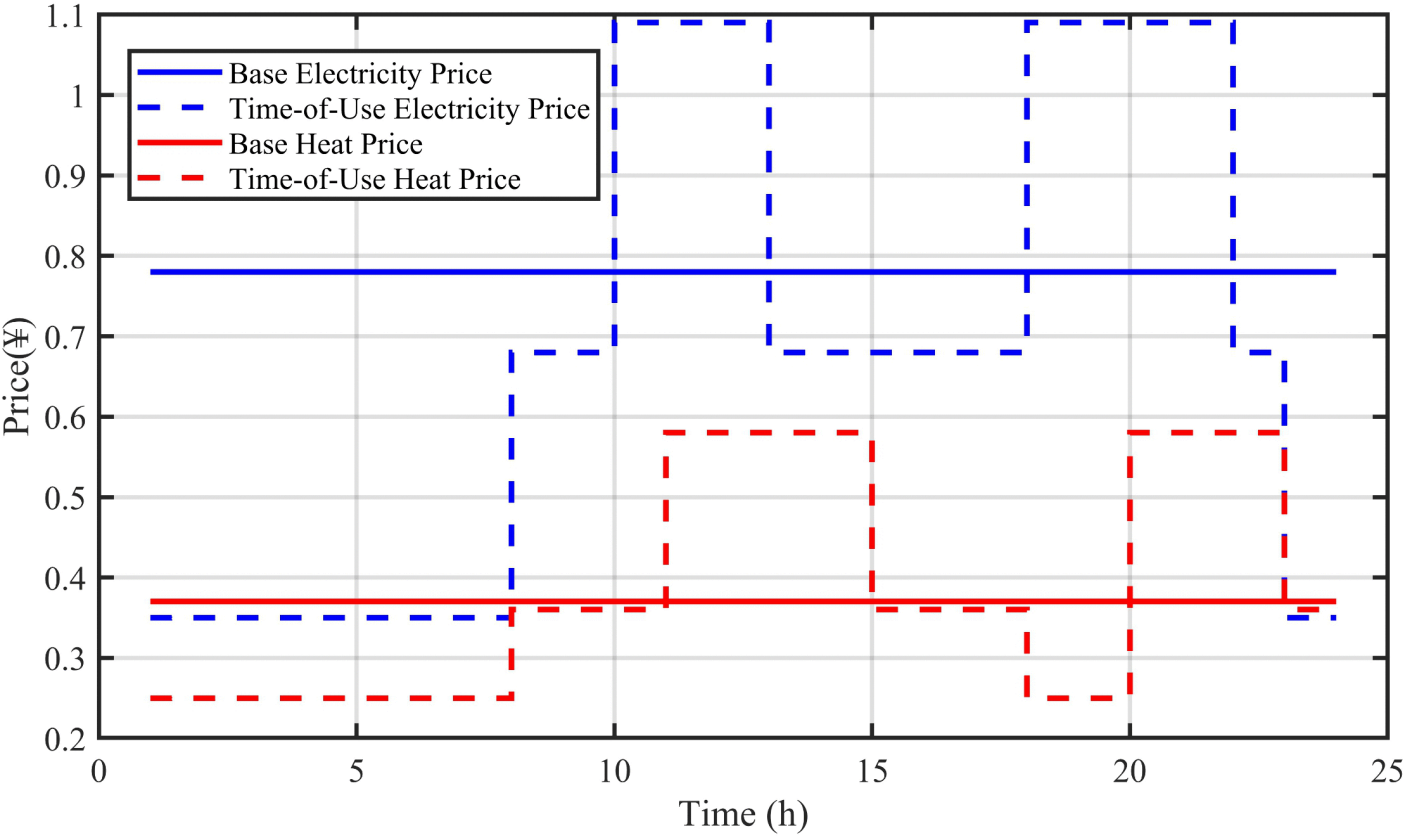
Energy price curves.

The operation behavior of the energy storage system shows a strong negative correlation with the energy prices in Fig 10, reflecting the classic economic arbitrage mode of “store low and discharge high”. As shown in Figs 10–12, the battery charges during nighttime electricity price valley periods (00:00–07:00) and discharges during afternoon and evening peak price periods. The SOC steadily increases during valley price periods and decreases during peak price periods, achieving the value of electricity time-shifting. The heat storage tank stores heat during early morning hours with low electricity prices and releases heat during high heat price periods in the afternoon and evening. Through heat storage configuration, the system effectively decouples the timing of “heat production” and “heat consumption”, improves the utilization of HP during low electricity price periods, and reduces overall heating costs.

**Fig 13:**
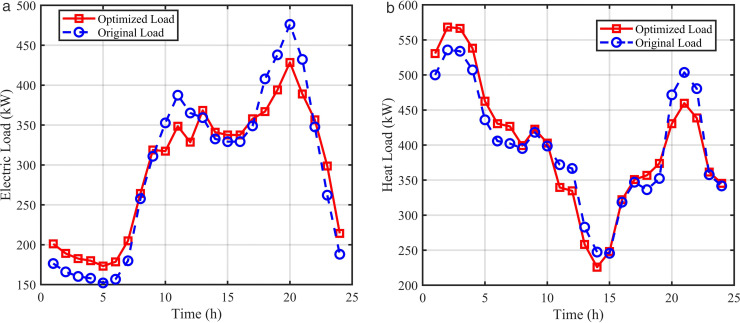
Comparison of consumer load curves before and after DR. (a) Electric load curve before and after DR, (b) Heat load curve before and after DR.

Fig 13 shows the electrical and thermal load curves before and after integrated demand response, respectively. The figures indicate that before demand response, the load curve has large peak-valley differences, while after demand response, the load curve becomes flatter. This is because after introducing the demand response mechanism, time-of-use electricity prices guide users to change electricity consumption habits and actively adjust usage periods, while also providing subsidies for load transfer and load interruption users, further encouraging participation in load adjustment.

Taking electrical load as an example, combined with time-of-use price information: during 10:00–12:00 and 18:00–21:00, the load reaches its peak while electricity prices are also at peak levels. Under the dual effects of price guidance and compensation incentives, some loads are interrupted during this period, while transferable loads are shifted to 01:00–09:00, 13:00–17:00, and 22:00–24:00. After implementing demand response, the electrical load achieves “peak shaving and valley filling”, reducing the peak-valley difference by 18.5% and effectively smoothing load fluctuations. The demand response process for thermal load is similar to that for electrical load.

**Fig 14:**
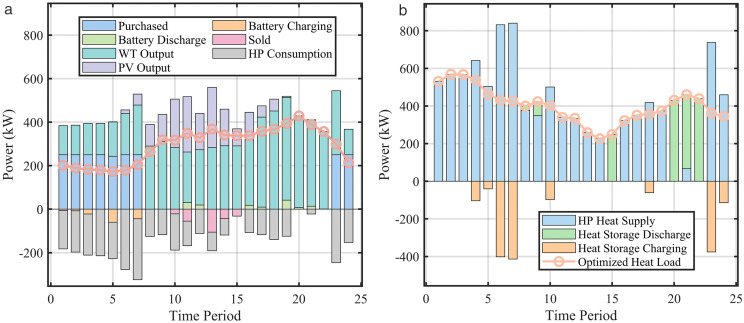
VPP balance after DR. (a) Electric load balance, (b) Heat load balance.

Fig 14(a) illustrates the power balancing results for electrical load after demand response. During 01:00–07:00, wind power primarily supplies the electrical load. Due to low electricity prices, the VPP purchases power from the main grid to charge batteries and converts electrical power to thermal power via HP to meet thermal load demand. From 08:00–21:00, PV power output is substantial, and electricity selling prices are high. After meeting load demand, the VPP sells surplus power to the main grid for additional revenue. Energy storage systems also discharge for arbitrage during this period. Moreover, due to high electricity prices, HP reduce electric-to-heat conversion power. During 22:00–24:00, electricity prices are low. To maintain consistent SOC at the start and end of the scheduling cycle, the VPP purchases power from the main grid to charge batteries and supplies heat to thermal loads via HP. Throughout the scheduling period, renewable generation dominates, with other dispatchable sources providing auxiliary regulation. GT contribute minimal generation because their operating costs exceed main grid purchase prices during valley price periods. During flat or peak price periods, abundant wind and solar power prioritize supply to loads, resulting in low gas turbine output.

Post demand response, the flattened load curve leads to smoother output profiles for DERs. Increased valley-period loads and reduced peak-period loads result in higher electricity purchases from the main grid during off-peak hours. Reduced peak loads during 08:00–21:00 enable greater electricity sales to the main grid, achieving arbitrage, increasing revenue, and enhancing the economic operation of the multi-energy VPP.

Fig 14(b) displays the power balancing results for thermal load after demand response. HP dominate the heat supply, while waste heat boilers and heat storage tanks contribute minimally, influenced by time-varying electricity/heat prices and equipment efficiency. During 01:00–10:00, HP operate at high capacity to meet most thermal load demand. Low electricity prices and adequate wind power supply allow bulk electricity purchases from the main grid for heating, simultaneously charging the heat storage tank. From 11:00–22:00, substantial electricity is sold to the grid for arbitrage, shifting thermal supply primarily to the heat storage tank and waste heat boilers. During 23:00–24:00, relatively low electricity prices prompt power purchases for HP operation and tank charging to maintain consistent stored heat levels.

### 5.3 Analysis of intraday rolling optimization results

**Fig 15:**
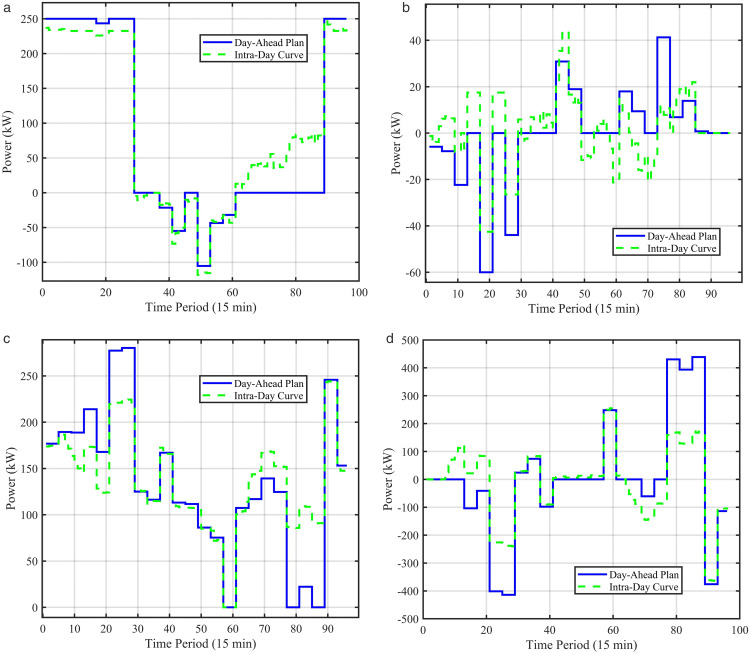
Intraday scheduling results. (a) Day-ahead vs intra-day VPP power exchange, (b) Day-ahead vs intra-day battery power, (c) Day-ahead vs intra-day HP power, (d) Day-ahead vs intra-day heat storage power.

Fig 15 compares the 15-minute intraday rolling scheduling results with the day-ahead hourly plan, showing the power adjustments of key equipment in the VPP during the intraday stage.

The comparison clearly indicates that the To overcome this fragmentation, tactual intraday scheduling strictly follows the overall trend of the day-ahead plan, attributed to penalty terms for deviations included in the intraday optimization objective function. However, within each rolling time domain, intraday scheduling dynamically fine-tunes operations based on more accurate 15-minute forecast data. If actual photovoltaic output exceeds the day-ahead forecast at any moment, the intraday scheduling may increase battery charging power or reduce grid purchases to utilize unexpected renewable generation in real time. Conversely, if actual load exceeds forecasts, the system responds by increasing energy storage discharge or grid power purchases.

### 5.4 Comprehensive performance evaluation

To quantitatively substantiate the claims made in this paper and provide the comparative benchmarks suggested, a comprehensive performance evaluation was conducted. This analysis deconstructs the proposed framework into its constituent components to rigorously assess the distinct and synergistic contributions of the advanced forecasting model and the dynamic demand response mechanism. For this purpose, three distinct operational scenarios were designed and simulated. To account for the stochasticity in model training, each scenario’s forecasting model was trained and evaluated 10 times with different random initializations. The key performance indicators (KPIs) derived from the dispatch simulations, averaged over the 10 runs, are systematically presented in Table 5. The first, designated as the baseline scenario (Case 1), utilizes a conventional LSTM model for load forecasting without the implementation of DR, representing a standard VPP operational approach. The second scenario (Case 2) integrates the proposed attention-augmented BiLSTM model for enhanced spatiotemporal load forecasting but continues to operate without the DR mechanism, thereby isolating the impact of forecasting accuracy. Finally, Case 3 represents the full implementation of our proposed integrated strategy, combining the high-precision forecasting model with the DDR framework.

**Table 5:** Performance comparison of different VPP optimization scenarios.

Performance Indicator	Case 1 (Baseline)	Case 2 (Enhanced Forecasting)	Case 3 (Proposed Strategy)
Forecasting Error (MAPE, %)	5.8 ± 0.9	2.5 ± 0.4	2.5 ± 0.4
Peak-Valley Load Difference (kW)	350 ± 25	350 ± 25	285 ± 21
Total Daily Operating Cost (k¥)	16.5 ± 1.2	15.3 ± 0.8	14.2 ± 0.7
Cost Reduction (vs. Baseline, %)	–	7.3 ± 1.5	13.9 ± 1.8
Renewable Curtailment (%)	4.5 ± 1.1	3.1 ± 0.8	1.8 ± 0.6

These metrics include forecasting accuracy, quantified by the Mean Absolute Percentage Error (MAPE), the effectiveness of load regulation, measured by the peak-valley load difference, and the overall economic efficiency, represented by the total daily operating cost and the associated cost reduction relative to the baseline. Furthermore, the technical benefit in terms of renewable energy integration is assessed through the renewable energy curtailment rate.

The results tabulated above provide clear, quantitative evidence of the proposed strategy’s superiority. A direct comparison between Case 1 and Case 2 elucidates the significant impact of the advanced forecasting model alone. By reducing the load forecasting MAPE from 5.8% to 2.5%, the VPP can achieve a more precise and proactive dispatch, resulting in a 7.3% reduction in total operating costs and a notable decrease in renewable energy curtailment. Subsequently, the introduction of the DDR mechanism, as demonstrated by the transition from Case 2 to Case 3, yields further profound benefits. The DR strategy effectively reshapes the load profile, achieving an 18.5% reduction in the peak-valley difference, which facilitates more economical energy arbitrage and better utilization of intermittent renewables. This culminates in the integrated strategy (Case 3) achieving a total operational cost reduction of 13.9% and halving the renewable curtailment rate compared to the baseline. This analysis confirms that a powerful synergistic effect emerges from the integration of high-fidelity spatiotemporal forecasting and adaptive demand-side management, validating the proposed framework as a more scientifically rigorous and economically efficient solution for modern VPP operation.

## 6 Conclusion

This study presented a comprehensive multi-timescale optimization strategy for VPP that leveraged AI-driven spatiotemporal load forecasting and DDR to enhance system operation. A novel attention-augmented BiLSTM model was developed for load prediction, which improved forecasting accuracy by fusing spatial and temporal features and provided a robust data foundation for VPP scheduling. The proposed MPC-based multi-timescale scheduling framework successfully adapted to RES uncertainties by optimizing both day-ahead and intra-day operations. Furthermore, the dispatch-integrated DDR strategy effectively miti-gated peak-valley load imbalances through real-time, closed-loop adjustments. The proposed framework’s effectiveness was validated through extensive simulations using a year-long set of actual operational data, which demonstrated its superiority over conventional approaches in reducing operational costs by 13.9%, improving load regulation, and enhancing overall grid stability. Our findings confirm that this integrated strategy offers a scientifically rigorous and practically viable solution for managing DERs in grids with high RES penetration.

While the proposed framework demonstrates significant advantages, future work can extend its capabilities in several key directions. One promising avenue is the integration of advanced AI control methods, such as deep Reinforcement Learning (RL), with the MPC framework. An RL agent could learn optimal control policies over time, dynamically adjusting MPC parameters to better handle non-stationary uncertainties that are difficult to model explicitly. In parallel, the demand response modeling could be significantly enhanced by moving beyond the current price-elasticity model to develop data-driven, agent-based models that capture the heterogeneous and uncertain behaviors of individual consumers, thereby allowing for more precise mobilization of demand-side flexibility. To further bolster the framework’s resilience, the deterministic optimization could be extended to a stochastic or robust formulation. By incorporating probabilistic forecasts directly from the AI model, the VPP scheduling could explicitly manage the risk associated with forecast errors. Finally, addressing the practical challenges of scalability and real-world validation is crucial for deployment. This includes investigating decentralized optimization algorithms to manage large-scale VPP and validating the entire strategy on a hardware-in-the-loop platform or in a pilot project to assess its performance against constraints like communication delays and imperfect data.

## Supporting information

S1 File The minimal data set. (RAR)
